# A multicenter real-world evidence study in the Swiss treatment landscape of chronic myeloid leukemia

**DOI:** 10.1186/s12885-022-10241-y

**Published:** 2022-11-19

**Authors:** Nathan Cantoni, Roberto Sommavilla, Patrick Seitz, Elisabeth Kulenkampff, Stefan Kahn, Jean-François Lambert, Adrian Schmidt, Reinhard Zenhaeusern, Stefan Balabanov

**Affiliations:** 1 Kantonsspital Aarau, Aarau, Switzerland; 2 Novartis Pharma Schweiz, Rotkreuz, Switzerland; 3 Hôpital de Nyon, Nyon, Switzerland; 4 Stadtspital Zürich Triemli, Zürich, Switzerland; 5 Spitalzentrum Oberwallis, Brig, Switzerland; 6 Department of Medical Oncology and Hematology, University Hospital Zurich and University of Zurich, Zürich, Switzerland

**Keywords:** Chronic myeloid leukemia, Deep molecular response, Real-world evidence, Tyrosine kinase inhibitors

## Abstract

**Background:**

The real-world experience of Swiss chronic myeloid leukemia (CML) patients treated with tyrosine kinase inhibitors (TKIs) is largely unknown, in particular with regard to achievement of response per European Leukemia Net (ELN) criteria and adherence to ELN recommendations.

**Methods:**

This was a retrospective, non-interventional, multicenter chart review of patients with newly diagnosed CML who had received first-line TKI and were solely treated with TKIs between 2010 and 2015, with a minimum follow-up of 18 months, at six Swiss hospitals. Effectiveness was evaluated according to ELN 2013 milestone achievements at 3, 6, 12 and 18 months, and at last follow-up.

**Results:**

Data from 63 patients (56% men; median age at diagnosis 55 years) were collected (first-line imatinib [*n* = 27], nilotinib [*n* = 27], dasatinib [*n* = 8], or ponatinib [*n* = 1]). TKI switches (49 times) and dosing changes (165 times) due to intolerance or insufficient response were frequent. Compared with patients receiving first-line imatinib, a higher proportion of patients receiving first-line nilotinib or dasatinib achieved optimal response at all timepoints, irrespective of subsequent TKI therapy, and a higher proportion of patients treated with first-line nilotinib and dasatinib reached deep molecular response (BCR-ABL1^IS^ ≤ 0.01%) at 18 months (42 and 38%, respectively, versus 27%). Patients who received nilotinib or dasatinib switched therapies less frequently than patients treated with imatinib, irrespective of subsequent TKI therapy.

**Conclusions:**

Although patient numbers were small, this real-world evidence study with patients with CML confirms that ELN guidelines are generally implemented in Swiss clinical practice, with a large proportion of patients achieving ELN 2013 milestones. While TKI use involved all inhibitors approved at the time of the study, an unexpectedly high number of TKI therapy switches suggests a clear difference in TKI use between registration trials and clinical practice.

**Supplementary Information:**

The online version contains supplementary material available at 10.1186/s12885-022-10241-y.

## Background

BCR-ABL1 tyrosine kinase inhibitors (TKIs) have revolutionized the therapy landscape of chronic myeloid leukemia (CML) and their efficacy has been well established in clinical trials [1–8]. However, CML management with TKIs in the context of patient care outside of clinical trials is complex. The correlation between results from clinical trials with TKIs and outcomes in a real-world setting in Switzerland has not been established previously.

Response to TKIs at key milestones is considered the most important prognostic factor. However, the proportion of patients achieving European LeukemiaNet (ELN)-defined milestones based on BCR-ABL1 mRNA levels [9–12] in routine clinical practice in Switzerland is unknown. Furthermore, physicians’ adherence to ELN recommendations in terms of CML management and the frequency of treatment switches in case of sub-optimal responses remain unclear, as registers and multicenter real-world data are not available.

To date, there are five TKIs approved for the treatment of chronic-phase CML in Switzerland: the first-generation TKI imatinib, the second-generation TKIs bosutinib, dasatinib, nilotinib and the third-generation TKI ponatinib. The more potent later-generation TKIs allow for greater reductions in the level of BCR-ABL1 mRNA [5, 8], which is prognostic for event-free survival, progression-free survival and overall survival in CML [1, 2, 13, 14].

Deep molecular responses (DMRs), i.e. ≥ 4-log (MR^4^) reduction of BCR-ABL1 transcript levels below the standardized baseline on the International Scale (BCR-ABL1^IS^), are discussed as a new goal of CML therapy [9, 15, 16]. DMRs are not only favorably associated with improved long-term outcomes but may also allow patients to interrupt or completely stop their treatment while remaining in a stable phase of molecular remission [16]. This concept, termed treatment-free remission (TFR), is currently evaluated in multiple clinical trials [17]. The ESMO guidelines for CML recommend TFR as a new treatment goal [15]; prerequisites for stopping treatment are close monitoring of the patient and durable achievement of at least MR^4^.

Here, we report findings from a non-interventional, multicenter, retrospective analysis aimed to describe the clinical routine and outcomes of TKI-based CML management since the introduction of second-generation TKIs in Switzerland.

## Methods

### Study design

The REVERT (**R**etrospective **EV**aluation of CML treatment in the **ER**a of second-generation **T**KIs) study was a multicenter, single arm, retrospective chart review with secondary use of data from CML clinical routine in Switzerland. Data were recorded between 23 November 2017 and 18 June 2018 at six sites in Switzerland (each with a minimum of five patients treated for CML) via a web-based electronic case report form. Participating sites were Universitätsspital Zürich, Kantonsspital Aarau, Hôpital Neuchâtelois, Stadtspital Triemli Zurich, Hôpital de Nyon, and Spitalzentrum Oberwallis. Adult patients (≥ 18 years) newly diagnosed with chronic phase CML between 1 January 2010 and 31 December 2015 and treated solely with TKIs were included in this study. Subsequent use of other TKIs was explicitly allowed. A minimum of 18 months follow-up was required, unless death occurred earlier. Supportive or temporary therapy (no longer than 3 months) with cytoreductive agents (e.g. hydroxyurea) before initiation of TKI therapy was allowed. Included were patients with either e13a2 (b2a2) or e14a2 (b3a2) transcripts, or both types; patients with atypical BCR-ABL1 transcripts were excluded.

### Participant recruitment and consent

Written informed consent was obtained from all individual participants before data collection. Data from patients deceased at the time of data collection were recorded only if previous consent had been given or if it had been obtained from a legal representative or a next of kin. All data was stored in an irreversible anonymized form. This study was performed in accordance with local laws and regulations as well as the Guidelines for Good Pharmacoepidemiology Practices of the International Society for Pharmacoepidemiology (ISPE 2008) [18], the STROBE (Strengthening the Reporting of Observational Studies in Epidemiology) guidelines [19], the ICH-GCP (International Conference on Harmonization of Good Clinical Practice) guideline [20], and with the ethical principles laid down in the Declaration of Helsinki.

### Endpoints and response definitions

The primary endpoint of this study was the proportion of patients achieving an ELN 2013-defined optimal treatment response at 3 months (BCR-ABL1^IS^ ≤ 10%), 6 months (BCR-ABL1^IS^ ≤ 1%), 12 months (BCR-ABL1^IS^ ≤ 0.1%), 18 months (BCR-ABL1^IS^ ≤ 0.1%) and at last follow-up (BCR-ABL1^IS^ ≤ 0.1%) [11]. ELN 2013 guidelines were the accepted standard at the time of the study, but have been updated in 2020 [9]. Secondary endpoints included the proportion of patients achieving DMR at any timepoint, defined as MR^4^ (BCR-ABL1^IS^ ≤ 0.01%) or MR^4.5^ (BCR-ABL1^IS^ ≤ 0.0032%), as well as the proportion of patients achieving a major molecular response (MMR), defined as BCR-ABL^IS^ ≤ 0.1%, at last visit. Further aims were to describe the TKI treatment landscape in Switzerland (first line [1 L] TKI usage, average duration of treatment, the frequency of and reasons for therapy changes, and frequency of inclusion in clinical trials), the adherence to ELN recommendations with respect to treatment changes and the evolution of BCR-ABL1 monitoring according to the IS (proportion of patients with BCR-ABL1^IS^ results per year).

### Statistical analyses

Data analysis was performed by descriptive statistics. For continuous variables summary statistics including mean, median, standard deviation, 1st quartile, 3rd quartile, minimum, maximum, and number of observations were used. For categorical variables, frequency counts and percentages were used. If appropriate, 2-sided 95% confidence intervals were derived. Kaplan-Meier estimators were derived for time-to-event analyses and for the estimation of failure/response rates at predefined time points (i.e. 3, 6, 12, 18 months, last follow-up). Statistical significance was defined as *p*-value ≤0.05 for all statistical test results. Box plots follow the classical methods of Tukey. Start of time intervals was the first administration of TKI.

## Results

### Patients

Data from 63 patients with CML treated with TKIs at Universitätsspital Zürich (*n* = 22), Kantonsspital Aarau (*n* = 16), Hôpital Neuchâtelois (*n* = 7), Stadtspital Triemli Zürich (*n* = 6), Hôpital de Nyon (*n* = 6) and Spitalzentrum Oberwallis (*n* = 6) were included in this study. Patients were observed for a median of 3 years (range, 2–5 years); during the observation time of this study, five patients deceased. The median time from CML diagnosis to therapy start with a TKI was 13 days (IQR, 7 to 21 days). At baseline, 56% of patients were men, and the median age at diagnosis was 55 years (IQR, 40 to 68); baseline characteristics were not significantly different between the TKI groups (Table 1). TKI therapy was most frequently initiated with nilotinib (*n* = 27) and imatinib (*n* = 27; including generic imatinib), while dasatinib (*n* = 8) and ponatinib (*n* = 1) were less frequently chosen in a 1 L setting. No patient received bosutinib in 1 L, which was not approved for 1 L treatment at the time. Most patients received the respective TKI at standard doses. Further baseline patient characteristics such as Sokal scores and transcript types are shown in Table 1*.* Progression to accelerated phase/blast crisis occurred in 3 of 63 (4.8%) patients. Five patients (8%) died as a direct or indirect consequence of CML (see Table S1 in the Supplementary Information).

**Table 1:** Baseline patient characteristics (ITT population)

1 L treatment^a^ ITT	Imatinib *N* = 26	Nilotinib *N* = 27	Dasatinib *N* = 8	Ponatinib *N* = 1	Imatinib (gen) *N* = 1	Total *N* = 63
Men, n (%)	13 (50)	15 (56)	6 (75)	1 (100)	0 (0)	35 (56)
Median age at diagnosis, years (IQR)	60 (40–75)	45 (38–65)	56 (50–61)	74	24	55 (40–68)
Sokal risk group at diagnosis, %						
Low	15	26	0	0	0	N.A.
Intermediate	42	26	63	100	0	
High	27	37	38	0	0	
Unknown	15	11	0	0	100	
Median Sokal score at diagnosis, score (IQR), *n*	1.04 (0.92–1.43), *n* = 19	1.16 (0.83–1.46), *n* = 16	1.14 (0.86–1.83), *n* = 6	– , *n* = 0	– , *n* = 0	1.10 (0.88–1.46), *n* = 41
Transcript type, *n* (%)						
e13a2 (b2a2)	10 (38)	9 (33)	2 (25)	0 (0)	0 (0)	21 (33)
e14a2 (b3a2)	12 (46)	9 (33)	5 (63)	1 (100)	0 (0)	27 (43)
both	3 (12)	1 (4)	1 (12)	0 (0)	0 (0)	5 (8)
unknown	1 (4)	8 (30)	0 (0)	0 (0)	1 (100)	10 (16)
Inclusion in clinical trial, *n* (%)	1 (4)	7 (26)	0 (0)	1 (100)	0 (0)	9 (14)
Median daily dose, mg (IQR)	400 (400–400)	600 (600–600)	100 (100–100)	45	400	–

There were no statistically significant differences between the groups for sex, age at diagnosis and median Sokal score (*p* > 0.05)

*IQR* interquartile range, *ITT* intention to treat population, *N.A.* not available

^a^No patient had been treated with 1 L bosutinib. The transcript type was determined by qualitative PCR

### Effectiveness

**Fig. 1:**
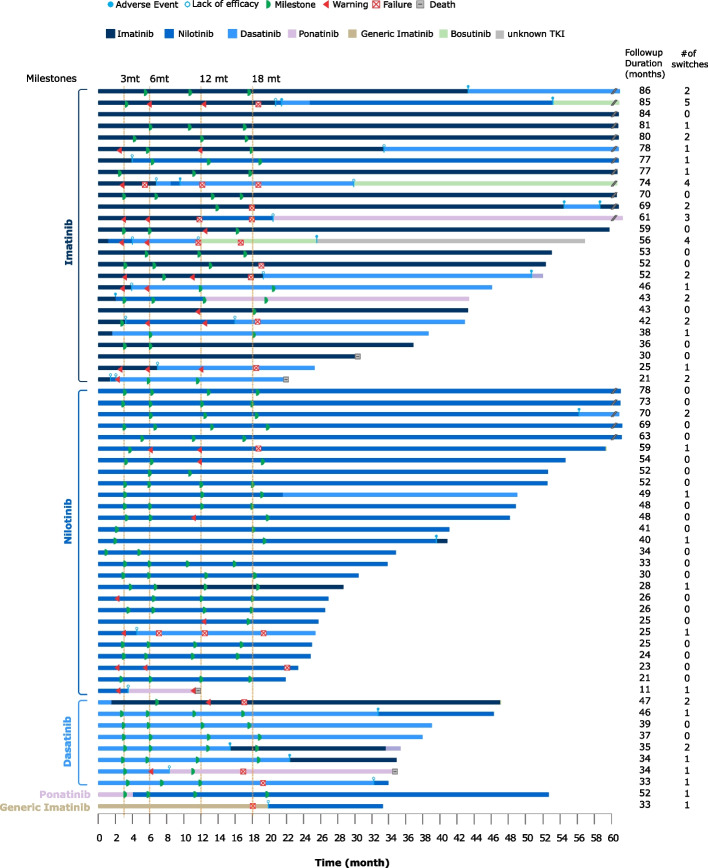
Treatment journey for each patient (*n* = 63), grouped by initial TKI and duration of follow-up duration. Sixty months of follow-up are shown; the total duration of follow-up and number of switches is shown on the right. Each row represents a single patient. Vertical orange dotted lines indicate target timelines for milestones according to the most commonly used guidelines (at 3, 6, and 12 months for ELN, and 3, 6, 12 and > 18 months for ESMO guidelines). See also Table S2 in the Supplementary Information for a list of adverse events leading to TKI discontinuation

**Fig. 2:**
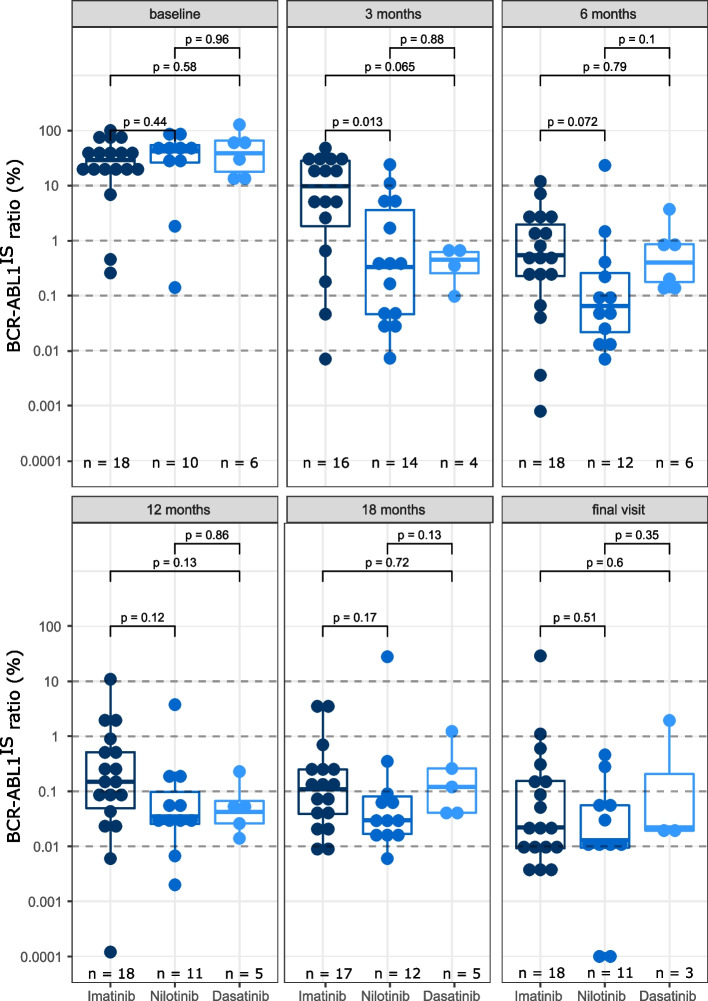
Response rates per BCR-ABL1^IS^ ratios in patients receiving imatinib (navy), nilotinib (azure) or dasatinib (light blue) as 1 L TKI.^a^ ^a^Only detectable BCR-ABL^IS^ ratios were included in this analysis. 1 L, first line; IS, International Scale; TKI, tyrosine kinase inhibitor

The treatment pathway for each patient (*n* = 63) demonstrated that TKI switches were very frequent throughout the study period (Fig. 1; Table S2 in the Supplementary Information shows adverse events leading to TKI discontinuation). For this reason, our analyses for response assessment are based on the ITT population of each TKI, i.e., the population of patients receiving a specified TKI as 1 L treatment, regardless of subsequent treatment switches. In order to evaluate the effectiveness in different groups we analyzed the response by BCR-ABL1^IS^ ratios per 1 L TKI at 3, 6, 12 and 18 months, and at final visit. For this analysis, data from patients treated with 1 L ponatinib and generic imatinib were excluded from the analyses owing to insufficient patient numbers (*n* = 1 each). At baseline median BCR-ABL1^IS^ ratios were similar across treatment groups (Fig. 2). After 3 months, patients treated with 1 L nilotinib reached a significant lower median BCR-ABL1^IS^ ratio compared with patients treated with imatinib (*p* = 0.013); the difference between imatinib and dasatinib groups at this timepoint was not significant. For the remaining time points the median BCR-ABL1^IS^ ratios in each group were not significantly different.

**Fig. 3:**
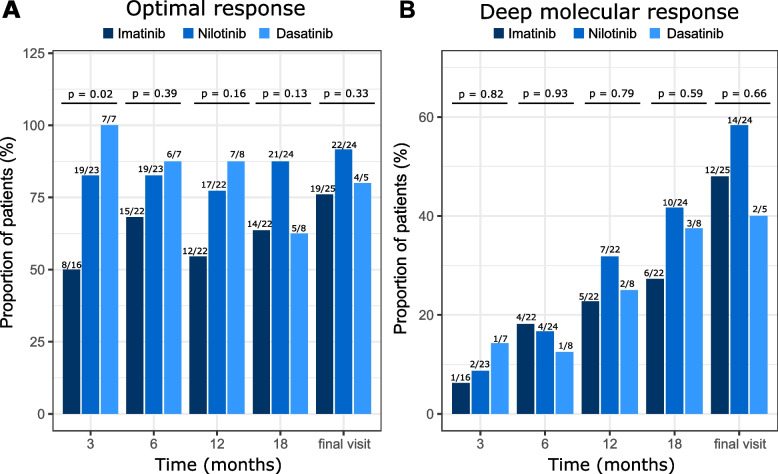
Proportion of patients reaching ELN 2013 milestones of (**a**) optimal response and (**b**) DMR (MR^4^ or deeper) at indicated timepoints per initial TKI. Numbers above bars represent patients that achieved optimal response/DMR per total patients with available response data at the respective timepoint in that treatment group. DMR, deep molecular response; TKI, tyrosine kinase inhibitor

We further analyzed the number of patients reaching ELN 2013 milestones of optimal response and DMR (MR^4^ or deeper) at 3, 6, 12, 18 months and at final visit (Fig. 3). 1 L treatment with nilotinib and dasatinib compared with imatinib consistently resulted in a higher proportion of patients achieving an ELN-defined optimal response at every timepoint (> 60% vs. ≥ 50%, respectively; statistical significance not reached); this difference was significant at the 3-month timepoint (imatinib vs. nilotinib vs. dasatinib, 50% [8 of 16 patients] vs. 83% [19 of 23 patients] vs. 100% [7 of 7 patients], respectively; *p* = 0.02; Fig. 3a).

A considerable proportion of patients in the imatinib (6 of 25 patients; 24%) and dasatinib (1 of 5 patients; 20%) groups had not achieved MMR (optimal response) at last visit. In contrast, only 2 of 24 patients (8%) of CML patients treated upfront with nilotinib did not reach MMR at the last follow-up visit (*p* = 0.33), despite these patients generally presenting with a higher Sokal score at baseline (see also Table 1). In total, 11 of 56 patients (20%) across all ITT groups did not reach MMR at final visit [this includes 2 patients from the generic imatinib and ponatinib arms (*n* = 1 each)].

**Fig. 4:**
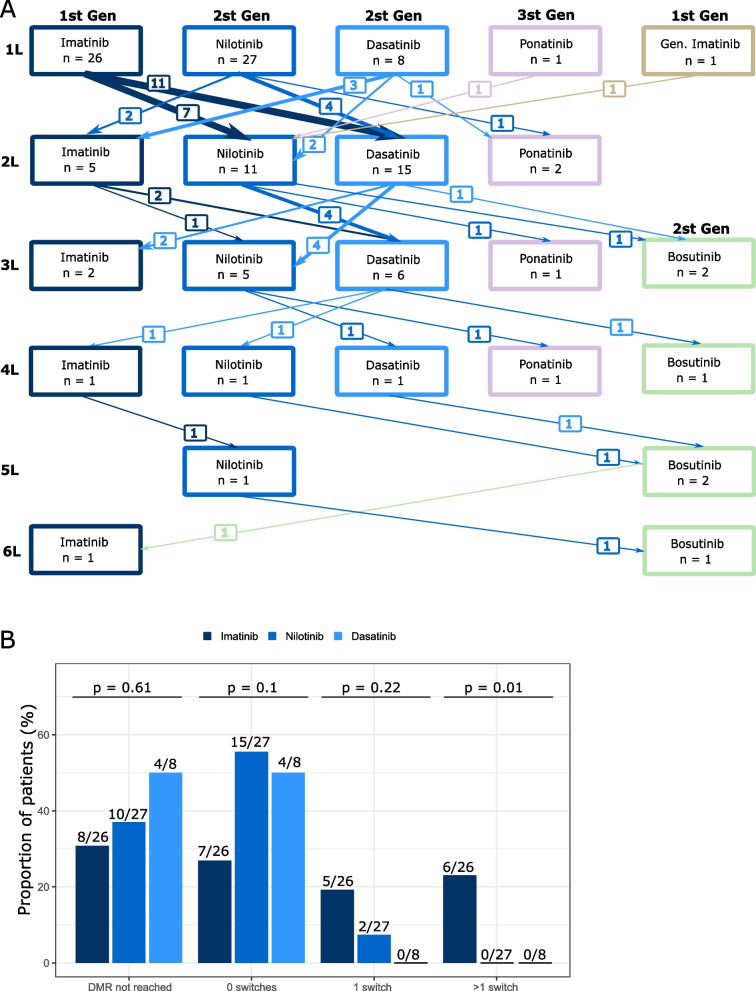
TKI switching. (**a**) TKI switch journey from 1 L to 6 L. Distribution based on initial TKI prescribed at diagnosis. (**b**) Number of switches until reaching DMR for the 3 most frequently used initial TKIs. Numbers above bars represent patients that achieved optimal response/DMR per total patients in that treatment group

A higher percentage of patients in the nilotinib and dasatinib versus the imatinib group reached DMR at 12 months (32% [7 of 22 patients] and 25% [2 of 8 patients], respectively, vs. 23% [5 of 22 patients]) and 18 months (42% [10 of 24 patients] and 38% [3 of 8 patients], respectively, vs. 27% [6 of 22 patients]; Fig. 3b). Note that 18 of 26 imatinib patients (69%) had switched to a second-generation TKI during the observation period, while only 7 of 27 patients who started on nilotinib (26%) switched TKI (see also Figs. 1 and 4A).

There was a trend for achieving MR^4.5^ more frequently with 1 L nilotinib: MR^4.5^ was observed in 23.9% of all PCR results (3-months to final visit measures) of patients treated with 1 L nilotinib; for 1 L imatinib and 1 L dasatinib, this was observed in 12.9 and 10.5%, respectively. However, this trend was not statistically significant (*p* = 0.2). The rate of achieving MR^4.5^ at any point during the treatment journey was similar for imatinib and nilotinib (nilotinib: 13 of 27 patients, 48%; imatinib: 11 of 26 patients, 42%), and lower for dasatinib (2 of 8 patients; 25%). The median time to first MR^4.5^ was 47 months for 1 L ponatinib (*N* = 1), 55 months for 1 L nilotinib (*N* = 27), and not reached for all other TKIs (data not shown). On-treatment achievement of MR^4.5^ was highest for nilotinib, where 13 of 43 patients (30%) ever-exposed achieved this milestone, compared with imatinib and dasatinib, where only 5 of 31 patients ever-exposed (16%) and 5 of 27 (19%) patients ever-exposed, respectively, reached on-treatment MR^4.5^ (*p* = 0.3).

### Response assessment

According to the ELN 2013 guidelines, 3-month, 6-month and 12-month qPCR and IS results are of key prognostic importance and should be recorded for every patient [21, 22]. Our results, however, show that 3-month IS results were available for only 47 of 63 patients (75%). This number was lower at all later timepoints: after 6 months, 12 months and 18 months, IS results were available for 36 of 63 patients (57%), 34 of 63 patients (54%), and 36 of 63 patients (57%), respectively. At the final visit, IS results were available for 37 of 63 patients (59%).

Mutations were very rarely detected in routine practice. One patient presented with a T315I/E255K mutation (1 L nilotinib group), which was detected as a result of the 3-month analysis.

### TKI switches

Frequent switches in TKI treatments were observed in the course of this study (see also Fig. 1). Of all 63 patients, 33 patients (52%) had received two or more lines of TKI treatment (Fig. 4a). Imatinib was used a total of 35 times, nilotinib 45 times, and dasatinib 30 times. We observed that intolerance of second-generation TKI was typically treated with dose reduction or change of TKI within the same class. On nine occasions patients treated 1 L with a second-generation TKI subsequently received imatinib (Fig. 4a).

During the study timeframe, 26 patients started 1 L therapy with the first-generation TKI imatinib (excluding generic imatinib), of which most patients (18 of 26, 69%) switched to a second-generation TKI (nilotinib or dasatinib), generally within 18 months of therapy start (see also Figs. 1 and 4A). This therapy change was due to imatinib intolerance in 5 patients, and due to suboptimal response in the remaining 13 patients.

A large proportion of patients did not reach DMR (MR^4^ or deeper) during the course of this study (50% [4 of 8 patients] for dasatinib; 37% [10 of 27 patients] for nilotinib; 31% [8 of 26 patients] for imatinib; *p* = 0.01; Fig. 4b). Analyzing the TKI treatment patterns of patients who reached DMR, we observed a difference in the number of switches between front-line treatments. A large proportion of patients achieved DMR while on treatment with frontline second-generation TKI, without treatment switch (56% [15 of 27 patients] in the nilotinib group and 50% [4 of 8 patients] in the dasatinib group); for imatinib, DMR without treatment switch was achieved less frequently (27% [7 of 26 patients]). In the population of patients that achieved DMR, 13 of 61 patients (21%) required at least one TKI switch before reaching DMR. Analyzing the number of switch events to reach DMR per TKI ITT, 5 of 18 imatinib patients (28%) and 2 of 17 nilotinib patients (12%) underwent one switch (Fig. 4b). Comparing first-generation to second-generation TKIs, a pattern of frequent switches before reaching DMR was observed in the imatinib group, where 11 of 18 patients (61%) had at least one switch before reaching DMR, while a single switch was sufficient to reach DMR in only 2 of 21 patients (14%) treated with second-generation TKI, despite the slightly higher median Sokal score of these patients at baseline (Table 1**,** Fig. 4b).

**Fig. 5:**
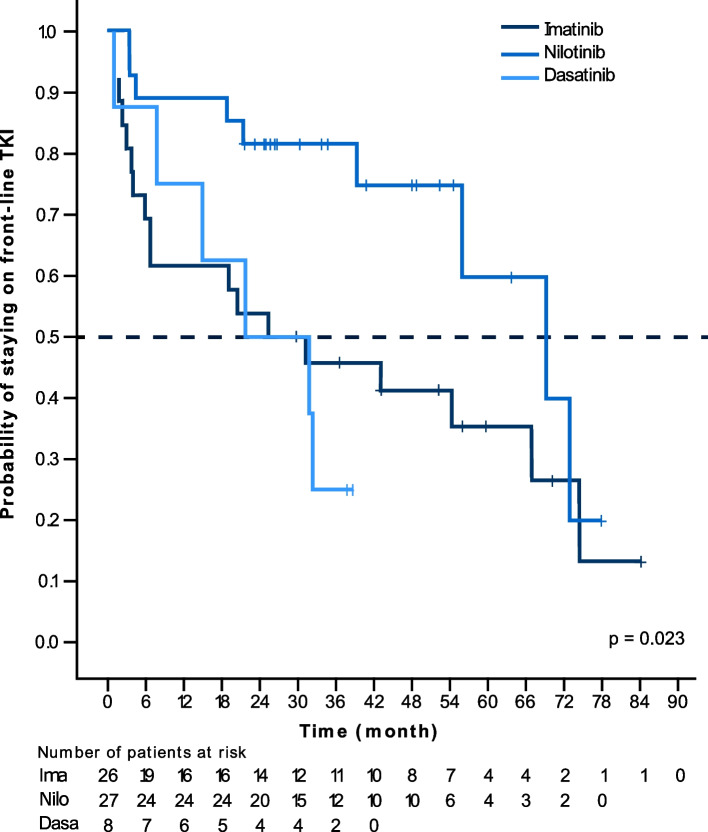
Kaplan-Meier curves for time on frontline TKI until first switch. Vertical lines indicate censored patients in each treatment arm

The proportion of patients remaining on their 1 L therapy throughout the study was significantly higher for patients starting on nilotinib (20 of 27; 67%; *p* = 0.002) compared with patients treated with 1 L dasatinib (2 of 8; 25%) or imatinib (8 of 26; 31%) (Fig. 1); this analysis excluded ponatinib and generic imatinib (1 patient each). The median duration of TKI treatment was higher for patients treated with nilotinib (24.0 months) and dasatinib (22.4 months) compared with imatinib (14.3 months; Table 2). Most patients treated with 1 L imatinib switched to another TKI (18 of 26 patients; 69%), after a median of 25.4 months (Table 2). A similar pattern was observed for 1 L dasatinib: 6 of 8 patients (75%) changed TKI drug, after a median of 22.2 months. However, most patients that received 1 L nilotinib stayed on treatment – only 9 of 27 patients (33%) switched to another TKI, and such a switch occurred after a median of 69.3 months. This difference in time-to-switch between nilotinib and imatinib as well as between nilotinib and dasatinib reached statistical significance (*p* = 0.023). At time 30 months (2.5 years) there was a 50% of probability of therapy switch for patients who started on imatinib or dasatinib, versus 20% for patients who started on nilotinib (Fig. 5).

**Table 2:** Treatment duration per TKI and median time to switch

	Imatinib *n* = 31	Nilotinib *n* = 43	Dasatinib n = 27	Ponatinib *n* = 5	Bosutinib n = 6	Generic imatinib n = 1
Median treatment duration, months (IQR)	14.3 (3.0–43.2)	24.0 (7.5–43.9)	22.4 (6.4–36.7)	8.4 (7.6–16.8)	4.8 (0.2–11.7)	20.3 (20.3–20.3)
*N* x months	812	1216	610	73.5	66	20.3
Total patient years	67	101	50.8	6.1	5.5	1.7
Median time to switch (estimate), months (95% CI)	25.4 (0.0–53.5)	69.3 (44.2–94.4)	22.2 (0.0–45.5)	4.0	N.A.	19.8

*IQR* interquartile range, *CI* confidence interval, *N.A.* not available

#### Frequency and reasons of therapy changes

Treatment changes (TKI switch, dose adjustment, dose interruption or re-initiation) were very frequent: a total of 165 changes were documented for 53 patients (84% of study population). Most of these changes occurred during 1 L treatment (96 changes 1 L vs 69 changes all later lines). The most TKI switches were observed in the imatinib (45% of changes) and dasatinib (52% of changes) ITT populations. In the nilotinib ITT population (41% of changes) therapy changes were mainly related to dose reductions (incl. TFR). Only few failures according to ELN guidelines were observed over the time course of follow-up (Fig. 1).

### Cardiovascular risk profiles

Risk factor recordings were missing for most patients in this study population; for this reason, cardiovascular risk could not be evaluated by risk scores. Information on documented risk factors, including smoking status, blood pressure and cholesterol levels is available in the supplementary information (Table S3 in the Supplementary Information).

### Adverse events

Clinical impactful adverse events that caused a change in therapy or dose for patients receiving imatinib, nilotinib and dasatinib are shown in Table 3 (due to limited patient information ponatinib, bosutinib and generic imatinib groups are not shown). In general, the type and distribution of documented adverse events matched the known profile of TKIs; no new safety signals were observed. Dasatinib had the highest frequency of events (45.2 events in 100 patient-years; Table 3). Imatinib was associated with slightly fewer adverse events than nilotinib (25.1 vs 30.6 events per 100 patient-years). However, it should be noted that patients in the nilotinib ITT population were younger and had higher Sokal scores at diagnosis than patients in the imatinib ITT population. Clinically relevant hematologic abnormalities were most frequent in the dasatinib group (21.6 events in 100 patient-years; compared with nilotinib [5.9 events in 100 patient-years] and imatinib [1.5 events in 100 patient-years]), while visceral organ adverse events (pancreatic, renal and liver events) were near-exclusive to nilotinib (8.9 events in 100 patient-years; compared with imatinib [1.5 events in 100 patient-years] and dasatinib [0 events in 100 patient-years]). Adverse events related to oedema/fluid retention and pleural effusion were more frequently observed for dasatinib versus nilotinib (11.8 vs 1 and 7.9 vs 0 events per 100 patient-years, respectively), whereas cardiovascular events were more frequent for nilotinib versus dasatinib (4.9 vs. 2 events per 100 patient-years).

**Table 3:** Adverse events that caused a change in dose or treatment switch for patients receiving imatinib, nilotinib and dasatinib

	Imatinib	Nilotinib	Dasatinib	Total TKIs^**a**^
**Any adverse event with clinical impact**	**17**	**31**	**23**	**79**
Events in 100 PTY	25.1	30.6	45.2	33.9
***Reason for dose adjustment/treatment switch, n***				
**Hematologic abnormalities**	**1**	**6**	**11**	**21**
Events in 100 PTY	1.5	5.9	21.6	9.0
**Gastrointestinal events**	**5**	**1**	**0**	**11**
Events in 100 PTY	7.4	1.0	0	4.7
**Pancreatic, renal, liver events**	**1**	**9**	**0**	**11**
Events in 100 PTY	1.5	8.9	0	4.7
**Oedema and fluid retention**	**3**	**1**	**6**	**10**
Events in 100 PTY	4.4	1.0	11.8	4.3
**Muscle skeletal joint**	**3**	**3**	**2**	**8**
events in 100 PTY	4.4	3.0	3.9	3.4
**Cardiovascular events**	**0**	**5**	**1**	**6**
Events in 100 PTY	0	4.9	2.0	2.6
**Skin, mucosal events**	**2**	**3**	**0**	**5**
Events in 100 PTY	3.0	3.0	0	2.1
**Pleural effusion**	**0**	**0**	**4**	**4**
Events in 100 PTY	0	0	7.9	1.7
**Pancreatic events**	**0**	**4**	**0**	**4**
Events in 100 PTY	0	3.9	0	1.7
**Hepatic events**	**0**	**3**	**0**	**4**
Events in 100 PTY	0	3.0	0	1.7
**Metabolism, homeostasis**	**1**	**2**	**1**	**4**
Events in 100 PTY	1.5	2.0	2.0	1.7
**Renal events**	**1**	**2**	**0**	**3**
Events in 100 PTY	1.5	2.0	0	1.3
**Other events of interest**	**0**	**1**	**2**	**3**
Events in 100 PTY	0	1.0	3.9	1.3

*PTY* patient years, *TKI* tyrosine kinase inhibitor

^a^also includes ponatinib and bosutinib events

## Discussion

This retrospective study provides unique insights into real-world treatment patterns and effectiveness of the five TKIs currently approved for the treatment of CML in Switzerland. Our observations confirm that TKI therapies are effective and tolerable in the majority of patients. However, patient treatment pathways were characterized by frequent dose adjustments and temporary treatment interruptions.

As expected in a real-world study, patient populations slightly differed from each other in terms of baseline characteristics, although these differences were not statistically significant. With a median age of 55 years, patients in this study were generally older than patients studied in the pivotal clinical trials, a phenomenon commonly observed for real-world data; for instance, baseline median age in the ENESTnd (nilotinib vs. imatinib) and DASISION (dasatinib vs. imatinib) trials was between 46 and 49 years [5, 8].

In line with findings from prospective, randomized controlled clinical trials that have demonstrated the superior efficacy of nilotinib and dasatinib versus imatinib [5, 8], we found a numerically higher chance for optimal treatment responses according to ELN 2013 guidelines associated with 1 L second-generation TKI treatment compared with 1 L first-generation TKI treatment. In addition, DMR was generally reached earlier in the nilotinib group than in the imatinib group (but differences between groups were not significant), despite 70% of imatinib patients switching to a second-generation TKI eventually. DMR has become an important milestone according to the latest ESMO guidelines for CML [15].

Interestingly, although second-generation TKIs were available during the study time, a large proportion of patients (41%) were treated with imatinib in the 1 L setting. With its well characterized safety profile and moderate toxicity, imatinib may be considered the frontline therapy of choice for patients with low risk scores, elderly patients and those with comorbidities, for which increased cardiovascular risks may limit the use of second-generation TKIs at therapy start. Second-generation TKIs were the preferred 1 L therapy for younger patients wishing to stop the TKI therapy once reaching a stable remission, or patients of childbearing age. This suggests that patient profiles, treatment goals as well as safety and efficacy aspects are taken into consideration by medical practitioners in Switzerland in order to identify an appropriate therapy.

TKIs have displayed high efficacy in clinical trials and high effectiveness in real-world studies; yet this study showed that a considerable proportion of patients (20%) has not reached MMR at last visit, and 8% of patients had deceased as a direct or indirect consequence of CML. These findings point to an important unmet medical need: despite a crowded market of TKI agents, the currently available agents may not be effective in a considerable proportion of patients. It is evident that new drugs are urgently needed for this patient population, ideally therapies with a different mode of action and/or against novel targets, such as immunotherapies. For example, asciminib, a BCR-ABL inhibitor with a novel mode of action, was granted US Food and Drug Administration (FDA) approval in October 2021 for patients with Philadelphia chromosome-positive CML in chronic phase and for adult patients with Philadelphia chromosome-positive CML in chronic phase with the T315I mutation [23]. Third generation TKIs may be soon reaching clinical routine, offering a new therapeutic option to patients not responding to currently approved TKIs.

We further noticed that most patients’ treatment journey was characterized by repeated switches in TKI, an observation previously described in real-world patients [24, 25]; the reason for this is unknown. Of the TKI inhibitors assessed in this study, nilotinib therapy was generally characterized by a lower number of switches and lower treatment duration. Most 1 L imatinib patients were eventually exposed to a second-generation TKI. The majority of switches occurred in the early stages of TKI therapy, suggesting a search for the optimal treatment, requiring TKI selection and doses that are able to provide the best combination of effectiveness and tolerability. The standard dosing of TKIs does not take into account the patient’s weight and pharmacokinetic aspects; therefore, treatment with the recommended dose may lead to overdosing and a possible higher incidence of adverse events, or underdosing, possibly resulting in an unsatisfactory response. It could be further hypothesized that some patients switched treatment at the appearance of low-grade adverse events, at much earlier times than it would be the case in a clinical trial setting. While it is possible to anticipate cardiovascular complications by weighing in certain risk factors as part of the patient assessment, it is difficult to predict some of the more severe side effects, such as muscle pain, oedema and metabolic, pancreatic and liver abnormalities. Adverse events that cause a reduction in the quality of life, but without potentially life-threatening consequences, might therefore be a trigger to switch TKI. In addition, the availability of five TKIs, of which most were approved not long before the start of the study, was opening a novel spectrum of possibilities for practitioners. Direct comparison of the efficacy and safety profiles of second-generation TKI has not been thoroughly explored in large phase 3 studies, and together with potentially limited experience with these drugs this might have contributed to the high number of treatment changes and switches, as there was no means of anticipating at therapy start the optimal therapy.

We found that on nine occasions patients treated with a second-generation TKI switched to first-generation imatinib, although such a therapy change is not described by the ELN recommendations [9, 11]. It could be hypothesized that physicians might have chosen a first-generation TKI after therapy failure with a second-generation TKI in patients with low risk scores or those with comorbidities due to the well-defined safety profile with fewer and milder adverse events.

The type and distribution of documented adverse events matches the known profile of TKIs described in the registration trials; however, 79 adverse events caused a clinical impact on the therapy, a phenomenon that is not captured in classic trials. Based on our real-world data the manifestation of adverse events may make physicians reconsider the chosen therapy and either suspend or adjust the TKI dose, or switch to another TKI altogether. Interestingly, in this study dasatinib had a higher frequency of clinically impactful adverse events than nilotinib and imatinib. Given that each patient had been exposed to more than 1 TKI it was not possible to further investigate the consequence of the sequential use of two or more TKIs in terms of adverse event causality.

Our findings further demonstrate that ELN recommendations are generally implemented in all participating Swiss centers. In particular, disease monitoring via BCR-ABL transcript measurement has become the gold standard over cytogenetic analysis during the study time. ELN 2013 recommendations were not universally implemented in some aspects, such as the use of imatinib after 1 L in 14% of patients, the incomplete cardiovascular risk assessment and the timing of PCR monitoring, which did not consistently take place in the ELN-recommended time windows.

This study had several limitations, including problems inherent to retrospective studies, such as lower levels of evidence compared with prospective studies. Although six Swiss centers contributed to this study, some of the larger study centers did not participate. Therefore, patient numbers were small, and effects may be over- or underestimated; conclusions have to be drawn with that in mind. Due to the small sample size, it was also not possible to demonstrate a significant difference between TKIs for many of the effectiveness endpoints. A further confirmatory study with a larger number of patients is warranted. Furthermore, the study was designed on the basis of the ELN 2013 guidelines. The 2020 version of the ELN guidelines have been updated in terms of assessment of risk status, place of the newer TKIs in treatment, patient monitoring, management of adverse events, the management of women who wish to become pregnant, and cost effectiveness [9]. However, this update does not affect the results and conclusions of this study. In addition, the ESMO 2018 guidelines as well as the ELN 2020 guidelines recommend treatment-free remission (TFR) as a new therapy goal [9, 15]. However, TFR was not captured consistently in this study as it was designed and performed before TFR became a treatment goal.

## Conclusions

In the dynamic field of CML therapy, TKI therapy is a successful treatment option in CML patients in routine clinical practice. Yet, a considerable number of patients does not respond adequately to all currently available TKIs, which highlights an existing unmet medical need in CML. The results presented in the REVERT study clearly evidence the effort in the clinical routine to identify the most appropriate therapy in terms of effectiveness and tolerability, opening the avenue of CML personalized medicine.

## Supplementary Information

**Additional file 1: Table S1.** Data on deceased patients and cause of death. **Table S2.** Data on adverse events leading to treatment change. **Table S3.** Data on available documented cardiovascular risk factors, including smoking status, blood pressure and cholesterol levels.

## Data Availability

The datasets generated during and/or analysed during the current study are available from the corresponding author on reasonable request.
