# Supplementary material for: A multicenter real-world evidence study in the Swiss treatment landscape of chronic myeloid leukemia
Source: BMC Cancer. 2022 Nov 19;22:1192. doi: 10.1186/s12885-022-10241-y (PMC9675134; doi:10.1186/s12885-022-10241-y)
Supplement: Supplementary file 1 — Additional file 1: Table S1. Data on deceased patients and cause of death. Table S2. Data on adverse events leading to treatment change. Table S3. Data on available documented cardiovascular risk factors, including smoking status, blood pressure and cholesterol levels. [file 12885_2022_10241_MOESM1_ESM.docx]

# A Multicenter Real-World Evidence Study in the Swiss Treatment Landscape of Chronic Myeloid Leukemia

Nathan Cantoni^1,^*; Roberto Sommavilla^2,^*; Patrick Seitz^2,^*; Elisabeth Kulenkampff^2^; Stefan Kahn^2^; Jean-François Lambert^3^; Adrian Schmidt^4^; Reinhard Zenhaeusern^5^; Stefan Balabanov^6,†^

^1^Kantonsspital Aarau, Aarau, Switzerland

^2^Novartis Pharma Schweiz, Rotkreuz, Switzerland

^3^Hôpital de Nyon, Nyon, Switzerland

^4^Stadtspital Waid and Triemli, Zürich, Switzerland

^5^Spitalzentrum Oberwallis, Brig, Switzerland

^6^Department of Medical Oncology and Hematology, University Hospital Zurich and University of Zurich, Zürich, Switzerland.

*These authors contributed equally to this work.

^†^Corresponding author: [stefan.balabanov@usz.ch](mailto:stefan.balabanov@usz.ch)

# Supplementary information

**Table S1. Survival status per baseline TKI**

|  | | **Imatinib** **N = 26** | **Nilotinib** **N = 27** | **Dasatinib** **N = 8** | **Ponatinib** **N = 1** | **Imatinib (gen.)** **N = 1** | **Total** **N = 63** |
| --- | --- | --- | --- | --- | --- | --- | --- |
| Deceased patients, n | | 2 | 1 | 1 | 0 | 1 | 5 |
| Cause of death, n | Acute prerenal renal failure (dehydration) and stop of supportive therapy (requested by patient) | 0 | 1 | 0 | 0 | 0 | 1 |
|  | Comorbidities and age | 1 | 0 | 0 | 0 | 0 | 1 |
|  | Progress | 0 | 0 | 1 | 0 | 0 | 1 |
|  | Septic shock due to severe graft-versus-host disease after stem cell transplant | 1 | 0 | 0 | 0 | 0 | 1 |
|  | Unknown | 0 | 0 | 0 | 0 | 1 | 1 |
| Death due to CML, n (%) | | 0 (0) | 1 (100.0) | 1 (100.0) | 0 (0) | 0 (0) | 2 (50) |
| Death due to study treatment, n (%) | | 0 (0) | 0 (0) | 0 (0) | 0 (0) | 0 (0) | 0 (0) |

CML, chronic myeloid leukemia; gen., generic.

**Table S2. Adverse events leading to treatment change, per baseline TKI**

| **Adverse event, n** | **Imatinib** | **Nilotinib** | **Dasatinib** | **Ponatinib** | **Generic imatinib** | **Total** |
| --- | --- | --- | --- | --- | --- | --- |
| Abdominal cramps, diarrhea | 1 | 0 | 0 | 0 | 0 | 1 |
| Acute renal insufficiency | 1 | 0 | 0 | 0 | 0 | 1 |
| Adverse effects, joint pain etc. | 0 | 0 | 2 | 0 | 0 | 2 |
| Back myalgia | 0 | 2 | 0 | 0 | 0 | 2 |
| Bilateral pleural effusion with dyspnea | 0 | 0 | 1 | 0 | 0 | 1 |
| Cerebral apoplexy | 0 | 2 | 0 | 0 | 0 | 2 |
| Cytopenia (platelets, neutrophils and anemia) | 1 | 0 | 0 | 0 | 0 | 1 |
| Cytopenia in the past | 1 | 0 | 0 | 0 | 0 | 1 |
| Diarrhea | 2 | 0 | 0 | 0 | 0 | 2 |
| E292K ABL1-Mutation | 1 | 0 | 0 | 0 | 0 | 1 |
| Epilepsy-like seizure | 1 | 0 | 0 | 0 | 0 | 1 |
| Erythema | 1 | 0 | 0 | 0 | 0 | 1 |
| Hematology toxicity: thrombocytes < 50x10^9^/L | 0 | 1 | 0 | 0 | 0 | 1 |
| Hepatopathy | 0 | 1 | 0 | 0 | 0 | 1 |
| Intolerance | 3 | 7 | 0 | 0 | 0 | 10 |
| Intolerance in the form of dyspnea | 0 | 1 | 0 | 0 | 0 | 1 |
| Large periorbital edema | 1 | 0 | 0 | 0 | 0 | 1 |
| Muscle cramps | 1 | 0 | 0 | 0 | 0 | 1 |
| Muscle pain | 1 | 0 | 0 | 0 | 0 | 1 |
| Pancreatitis | 0 | 2 | 0 | 0 | 0 | 2 |
| Pancytopenia | 1 | 1 | 0 | 0 | 0 | 2 |
| Peripheral artery disease with multiple recanalizations | 1 | 0 | 0 | 0 | 0 | 1 |
| Pericardial and pleural effusion with heavy night sweats | 0 | 0 | 1 | 0 | 0 | 1 |
| Pericardial effusion | 0 | 0 | 1 | 0 | 0 | 1 |
| Peripheral arterial disease | 1 | 0 | 0 | 0 | 0 | 1 |
| Pleural effusion | 1 | 0 | 0 | 0 | 0 | 1 |
| Pleural effusion, pulmonary emphysema | 1 | 0 | 0 | 0 | 0 | 1 |
| Pulmonary hypertension | 1 | 0 | 0 | 0 | 0 | 1 |
| Sensorimotor polyneuropathy | 0 | 1 | 0 | 0 | 0 | 1 |
| Severe cutaneous rash (exanthema) | 1 | 0 | 0 | 0 | 0 | 1 |
| Severe hypoglycemia | 1 | 0 | 0 | 0 | 0 | 1 |
| Subileus, nausea, vomiting | 1 | 0 | 0 | 0 | 0 | 1 |
| Symptomatic anemia | 0 | 0 | 1 | 0 | 0 | 1 |
| Thrombocytopenia (grade 3) | 0 | 1 | 0 | 0 | 0 | 1 |
| Thrombocytopenia and neutropenia | 1 | 0 | 0 | 0 | 0 | 1 |
| Toxic pancreatitis, elevated lipase | 0 | 1 | 0 | 0 | 0 | 1 |

TKI, tyrosine kinase inhibitor.

## Cardiovascular risk profiles

Second-generation TKIs are associated with increased vascular and cardiovascular events, and a thorough characterization of patient risk factors is recommended in order to reduce the morbidity and mortality associated with cardiovascular events [1, 2]. Risk factor recordings were missing for most patients in this study population; for this reason, cardiovascular risk could not be evaluated by risk scores. Available documented risk factors included smoking status (documented for 43 of 63 patients [68%]), blood pressure (documented for 37 of 63 patients [59%]), and cholesterol levels (documented for 10 of 63 patients [16%]; **Table S3).**

**Table S3. Cardiovascular risk factors.**

| **1L treatment** | **Imatinib** | **Nilotinib** | **Dasatinib** | **Ponatinib** | **Imatinib (gen.)** | **Total** |
| --- | --- | --- | --- | --- | --- | --- |
| Smoking, n (%)  yes  ex-smoker  no  unknown | 3 (11.5)  3 (11.5)  10 (38.5)  10 (38.5)  **n = 26** | 2 (7.4)  5 (18.5)  14 (51.9)  6 (22.2)  **n = 27** | 0 (0)  0 (0)  5 (62.5)  3 (37.5)  **n = 8** | 0 (0)  0 (0)  1 (100)  0 (0)  **n = 1** | 0 (0)  0 (0)  0 (0)  1 (100)  **n = 1** | 5 (7.9)  8 (12.7)  30 (47.6)  20 (31.7)  **N = 63** |
| Median total cholesterol, mg/dl (IQR) | 50.0 (0−141)  **n = 4** | 161.0 (144−201)  **n = 8** | -  **n = 0** | -  **n = 0** | -  **n =0** | 152.0 (120−179)  **n = 12** |
| Median systolic blood pressure, mmHg (IQR) | 137  (122−145)  **n = 19** | 124  (115−134)  **n = 12** | 126  (126−134)  **n = 5** | 144  (144−144)  **n = 1** | -  **n = 0** | 130 (120−142)  **n = 37** |

1L, first line; gen., generic; IQR, interquartile range.

**References:**

1. Barber MC, Mauro MJ, Moslehi J. Cardiovascular care of patients with chronic myeloid leukemia (CML) on tyrosine kinase inhibitor (TKI) therapy. Hematology Am Soc Hematol Educ Program 2017; 2017: 110-114.

2. Pagnano KBB, Assunção PM, Zullli R et al. Assessment of Cardiovascular Events in Chronic Myeloid Leukemia Patients Treated with Tyrosine Kinase Inhibitors. Blood 2015; 126: 4031.
